# Metabolomics: A Scoping Review of Its Role as a Tool for Disease Biomarker Discovery in Selected Non-Communicable Diseases

**DOI:** 10.3390/metabo11070418

**Published:** 2021-06-25

**Authors:** Adewale Victor Aderemi, Ademola Olabode Ayeleso, Oluboade Oluokun Oyedapo, Emmanuel Mukwevho

**Affiliations:** 1 Manchester Institute of Biotechnology, University of Manchester, Manchester M1 7DN, UK; adewale.aderemi@postgrad.manchester.ac.uk; 2 Department of Medical Biochemistry, Osun State University, Osogbo PMB 4494, Nigeria; 3 Department of Biochemistry, Adeleke University, Ede PMB 250, Nigeria; ademola.ayeleso@adelekeuniversity.edu.ng; 4 Department of Biochemistry, Obafemi Awolowo University, Ile-Ife 220282, Nigeria; ooyedapo@yahoo.co.uk; 5 Department of Biochemistry, Mafikeng Campus, North-West University, Mmabatho 2735, South Africa

**Keywords:** metabolomics, metabolites, biomarkers, analytical tools, diseases, diabetes, obesity

## Abstract

Metabolomics is a branch of ‘omics’ sciences that utilises a couple of analytical tools for the identification of small molecules (metabolites) in a given sample. The overarching goal of metabolomics is to assess these metabolites quantitatively and qualitatively for their diagnostic, therapeutic, and prognostic potentials. Its use in various aspects of life has been documented. We have also published, howbeit in animal models, a few papers where metabolomic approaches were used in the study of metabolic disorders, such as metabolic syndrome, diabetes, and obesity. As the goal of every research is to benefit humankind, the purpose of this review is to provide insights into the applicability of metabolomics in medicine vis-à-vis its role in biomarker discovery for disease diagnosis and management. Here, important biomarkers with proven diagnostic and therapeutic relevance in the management of disease conditions, such as Alzheimer’s disease, dementia, Parkinson’s disease, inborn errors of metabolism (IEM), diabetic retinopathy, and cardiovascular disease, are noted. The paper also discusses a few reasons why most metabolomics-based laboratory discoveries are not readily translated to the clinic and how these could be addressed going forward.

## 1. Introduction

Metabolomics is one of the latest on the list of ‘omics’ sciences after genomics, proteomics, and transcriptomics and combines high-throughput analytical techniques with bioinformatics. It deals with quantitative and qualitative assessments of metabolites, which are important intermediates and end products of metabolism [1]. The goal of this scientific approach is not just to decipher what pathological processes or perturbations underlie a given disease entity but also to predict responses of such conditions to therapeutic interventions. Metabolomic analyses allow us to distinguish between normal and pathological pathways, thereby helping in making disease diagnosis and predicting prognosis [2]. Given the fact that metabolites are a downstream expression of the various changes that occur in the genome, the proteome, and the transcriptome, they can closely represent the phenotypic fingerprints of an organism at any point in time. The totality of these small-molecule metabolites that are found in a biological sample at a given physiological period and that give an express functional summary of all the metabolic activities going on in a particular biological sample is termed metabolome [1,3,4]. One notable advantage of metabolome over genome is its ability to reflect the environmental influences [5] and to give a snapshot of the patho-physiological condition of the individual at a specific point in time [6]. In animal models, we have utilised a couple of these tools to study some metabolic disorders, such as metabolic syndrome, diabetes, and obesity [7,8,9]. Our goals in those studies were to gain better insights into the biochemical and pathological processes that are perturbed and to inform the development of more improved therapeutic agents in the management of those disease conditions in humans. Metabolomic tools offer the advantages of being fast, cheap, and sensitive. However, the data generated by these tools mean nothing unless they are properly analysed for the construction of biochemical pathways and for understanding how these pathways interact in both diseased and nondiseased states [10].

There are several approaches to metabolomics, each of which could be achieved by either mass spectrometry (MS), nuclear magnetic resonance (NMR) spectroscopy, or Fourier-transform infrared (FTIR) spectroscopy techniques (see Figure 2). Examples of such approaches include metabolomic fingerprinting, metabolic profiling, metabolic footprinting, target analysis, and flux analysis, each playing significant roles in understanding toxicological mechanisms and disease processes in living organisms [1,11,12]. Metabolomics is an equally valuable tool for new drug discovery; biomarker discovery for early disease diagnosis, such as diagnosis of rheumatoid or osteoarthritis [13,14,15,16], osteoporosis [17], cardiovascular disease [18], and Alzheimer’s disease (AD) [19,20]; monitoring of cancer prognosis, diagnosis, and treatment [20,21,22,23,24,25,26,27,28]; inborn errors of metabolism (IEM) [29]; and a host of others. Metabolomics also has applications in such areas as physiology, safety evaluation of new drugs, nutrition, and environmental assessment. The aim of this review is to give an overview of various metabolomic approaches and to highlight some recent biomarker discoveries for disease diagnosis, treatment, and prognosis using these analytical tools.

### 1.1. Metabolomic Processes/Workflow

**Figure 1:**
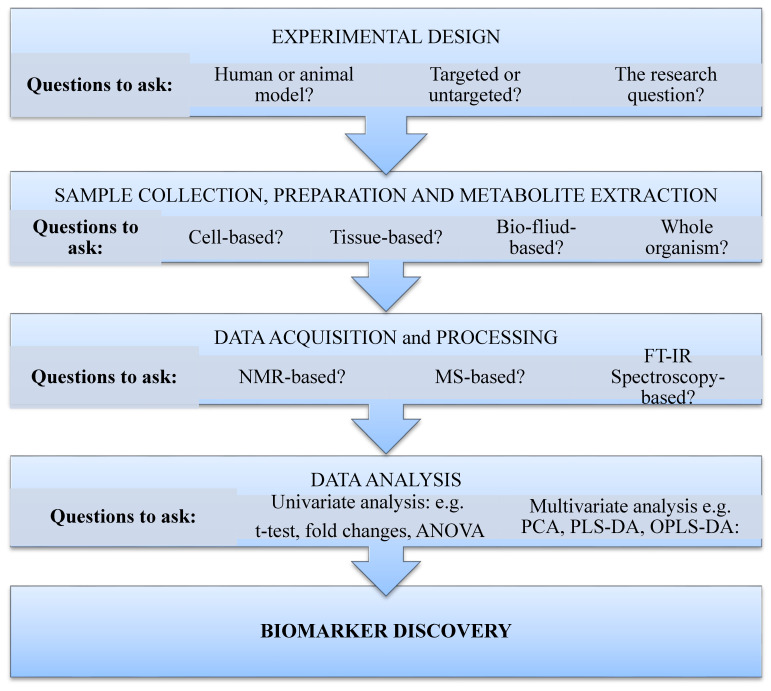
Metabolomic workflow. The first stage involves experimental design, followed by sample collection, preparation, and metabolite extraction. Next is acquisition and processing of data, then data analysis, and finally, making sense of the data through biomarker discovery.

For a successful targeted or untargeted metabolomic study, the following steps, which have been previously described elsewhere [30], are followed: experimental design; sample collection, preparation, and metabolite extraction; data acquisition and processing; statistical analysis; and biomarker discovery. Experimental design, the first step in the metabolomic workflow, is informed by the nature of the biological specimen to be used. Hence, questions such as whether human or animal samples or models will be used, whether it will be cell based, tissue based, the whole organism, fluid, or growth medium based, must be answered. Additionally, the metabolomic approach to be employed (targeted or untargeted) and the gap the study attempts to bridge or the problem it seeks to solve are important considerations in designing the experiment. Once this has been settled, the researcher moves to the next phase, namely, sample collection, preparation, and metabolite extraction. For an MS-based approach, this stage is often required and may involve liquid–liquid extraction, solid-phase extraction, or filtration technique, depending on the biological samples one is dealing with. For an NMR-spectroscopy-based approach, little or no sample preparation may be required. The third step involves data acquisition and processing. This involves the use of analytical platforms such as MS, NMR, and FTIR spectroscopy. The fourth step is statistical analysis. Here, either or both a univariate analytical tool (e.g., *t*-test, ANOVA, fold changes) and a multivariate analytical approach (i.e., use of PCA, PLS-DA, OPLS-DA) could be employed. The last step is biomarker discovery. Here, metabolites with differential expressions (overexpression or downregulation) are identified using standardised bioinformatics tools and databases. Figure 1 below gives a summary of the processes involved and the research questions to ask for a successful metabolomic study.

### 1.2. Analytical Platforms for Data Acquisition and Processing: Strengths and Limitations

**Figure 2:**
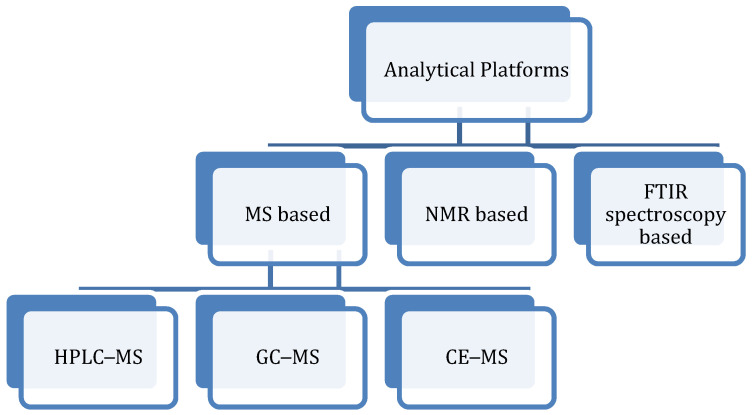
Summary of the major analytical platforms for metabolomic studies in human and animal samples. The figure depicts the three most often employed platforms for metabolomic studies—MS, NMR, and FTIR spectroscopy. The MS-based approach involves coupling with liquid chromatography (LC), gas chromatography (GC), or capillary electrophoresis (CE).

To have a better understanding of the metabolic milieu of biological systems, analytical platforms are employed to identify and quantify small-molecular-weight metabolites ahead of further analysis of the data generated by the platforms. The two most predominantly utilized analytical tools for metabolomic studies are NMR and MS spectroscopy [1,31]. The MS approach is usually coupled with chromatographic techniques, such as liquid chromatography (LC–MS), especially high-performance liquid chromatography (HPLC–MS), and gas chromatography (GC–MS) [8,32] with varying degrees of sensitivity. The MS technique can also be coupled to capillary electrophoresis (CE–MS). Although NMR offers such benefits as easy sample preparation, shorter time for sample analysis, easily identifiable metabolites from analysis of spectra, and better sample recovery, it is only able to analyse less variety of metabolites, and only a few comprehensive metabolite databases for NMR-based metabolomics are currently available [6]. The ease of sample preparation and the reproducibility of result have made NMR spectroscopy, therefore, a highly sought-after approach for the structural analysis of metabolites [33]. On the other hand, MS-based techniques can analyse a wide range of metabolites following chromatographic separation, and several comprehensive metabolite databases are presently available [17]. Nonetheless, the sensitivity of MS-based platforms depends on the chromatographic separation technique with which it is combined [33]. For instance, while GC–MS gives better chromatographic resolution of the metabolites, LC–MS is the preferred combination when it comes to metabolite coverage and sensitivity [33,34,35,36]. The MS approach is, however, not without its drawbacks, namely, the sample preparation is more rigorous, the technique could fragment the samples, making recovery almost impossible; and there is much difficulty identifying unknown compounds on its spectra [6]. Metabolite identification and quantification using the NMR approach (e.g., proton NMR, ^13^C NMR, ^19^F NMR, ^31^P NMR spectroscopy) have been improved in recent years. Of note is the introduction of cyroprobes and microprobes, which have reduced the detection limit by a factor of about 3 to 5 [18]. This approach is further enhanced by using two-dimensional total correlation spectroscopy (2D TOCSY) for the confirmation of assigned peaks. The 2D TOCSY spectrum usually shows the correlations between two frequency axes that are derived from two-dimensional Fourier transformations. Nuclear Overhauser effect spectroscopy (NOESY), heteronuclear single quantum coherence (HSQC), exchange spectroscopy (ES), and J-spectroscopy (JS) are a few other examples of two-dimensional NMR that have been used to improve NMR-based data acquisition and metabolite structure analysis, thereby providing better information than one-dimensional NMR, especially for small-molecule metabolites [37,38,39]. Additionally, better outcomes can be achieved by combining two forms of 2D NMR, such as NOESY and HSQC, TOCSY, and HSQC; by combining MS with NMR spectroscopy [40]; or by using three-dimensional NMR methods. Apart from NMR and MS platforms, another tool that is also being increasingly utilised for metabolomic studies is FTIR spectroscopy [32]. In general, identification of metabolites is based on their mass, mass/charge ratios, and retention time [6]. With these parameters in place, both known and unknown compounds could be identified by comparing them with metabolite databases, such as Metlin/XCMS and the Human Metabolome Database [6]. For compounds with unknown MS and NMR spectra characteristics, an untargeted approach and chemometrics could be applied for metabolite pattern identification [6]. A brief description of the foremost analytical platforms for metabolomic studies is shown in Figure 2.

### 1.3. Metabolomic Data Analysis

Profiling the metabolites in each biological entity is incomplete without an accurate data measurement and precise interpretation of the information garnered from such exercise. Metabolomic data analysis encompasses feature extraction, compound identification, statistical analysis, and interpretation. Use of multivariate analyses, such as PCA, PLS-DA, and OPLS-DA, is key to achieving this. Together, these pattern recognition analytical techniques (PCA, PLS-DA, and OPLS-DA) help to comprehensively assess the metabolites that are present in any given biological sample or that are associated with a specific disease condition [11]. Scores from PCA plots show a scattering of the samples, and when they are clustered together, it shows that the metabolites are alike; otherwise, they are dissimilar [11]. PLS-DA, on the other hand, is a very versatile algorithm with a better predictive and descriptive advantage over PCA. It seeks to maximise the covariance between the classes much better than PCA. Bioinformatics tools available for metabolomic data analysis, pathway analysis, and interpretation include Metabox (available for free at http://kwanjeeraw.github.io/metabox/ (accessed on 10 January 2020) under the GPL-3 license) [30] and MetaboAnalyst (available at http://www.metaboanalyst.ca/MetaboAnalyst (accessed on 10 January 2020)) [20]. Others include SECIMTools, Meta XCMS, XCMS, XCMS2, MetAlign, MZmine for data processing of MS data, and MetDAT for statistical analysis and pathway visualization.

## 2. Results

This mini review focuses on five disease conditions in which metabolomic tools have been utilised for diagnosis, treatment, or prognosis prediction. The first four of these conditions are chronic non-communicable diseases in adults, while the fifth is a group of inborn errors of metabolism in children. These medical disorders are Parkinson’s disease (PD), diabetic retinopathy, Alzheimer’s disease (AD)/dementia, cardiovascular disease, and inborn error or metabolism (IEM). Out of the 26 publications that were included in this review, 3 were adjudged to be relevant to our research interest with respect to PD [41,42,43], 5 with respect to diabetic retinopathy [30,44,45,46,47], 2 for AD [16,46], 14 for cardiovascular disease [18,48,49,50,51,52,53,54,55,56,57,58], and 2 for IEM [59,60]. Of the 3 most often utilised platforms for metabolic profiling, MS spectroscopy (coupled with other separation techniques) ranks first, being employed for biomarker discovery in 17 out of the 26 studies identified, and accounting for 65.4% of all studies. This was followed by NMR spectroscopy, which accounted for just 19.2% (5 studies) of cases. In terms of statistical analysis, multivariate analysis involving PCA and/or PLS-DA was utilised in 6 studies [41,46,47,49,54,61], followed by the use of receiver operating characteristic (ROC) curve or area under the curve (AUC) analysis [30,42,50,56,58]. Details of the study characteristics, the analytical tools employed, and the respective biomarkers identified are summarised in Table 1 and Table 2, respectively.

## 3. Discussion

A combination of metabolomic study and multivariate data analysis offers tremendous advantages of understanding specific pathways of metabolism that are perturbed in a particular disease state [10,16]. Information derived from such scientific efforts goes a long way in providing insights into useful diagnostic and therapeutic metabolite biomarkers for effective disease management [14] and prognostication. Below are a few examples of disease conditions with their recently documented clinically relevant metabolic biomarkers.

### 3.1. Parkinson’s Disease

Currently, PD affects well over 4 million people globally, and sadly, this figure is expected to double over the next few decades [65]. This progressive neurodegenerative disorder, affecting mostly the adult population, is difficult to manage. Despite many years of research, a lot is still unknown about the aetiology of the disease [66]. The reason for this may be that it is a multifactorial disease, and that multiple mechanistic pathways may be involved in its causation [21]. In addition, only a few disease-modifying therapies are presently available. This is largely secondary to absence of effective biomarkers that could aid in both disease diagnosis and treatment [66]. Existing diagnosis efforts rely heavily on symptoms, patient history, and clinical examination, making misdiagnosis inevitable in clinical settings [66]. A better diagnostic approach that is simple, fast, and less invasive is therefore needed, and metabolomic tools have been reported to show great assurance in this regard [41]. In a recent study, Saiki et al. [42] carried out a metabolomic analysis using capillary electrophoresis and liquid chromatography coupled with MS on blood samples (a less invasive procedure compared with the use of CSF) and identified 18 PD-specific metabolites. In this research, significant decreases in the levels of seven long-chain acylcarnitines were identified as promising metabolite biomarkers in PD diagnosis. In another study, Havelund et al. [43] utilised LC–MS to assess levels of kynurenine metabolites in both plasma and cerebrospinal fluid samples of healthy individuals and compared them with those of three categories of PD patients: PD patients not on medications, PD patients undergoing treatment using L-3,4-dihydroxyphenylalanine (L-DOPA) who have developed dyskinesia, and PD patients who are yet to develop dyskinesia despite prolonged use of L-DOPA. Their findings showed an approximate fourfold rise in the ratio of 3-hydroxykynurenine to kynurenic acid in plasma samples and a significant decrease in anthranilic acid levels in both plasma and CSF samples of PD patients with L-DOPA-induced dyskinesia. The study further reported a twofold increase in the levels of 5-hydroxytryptophan in all of the L-DOPA-treated PD patients (with or without dyskinesia). The researchers concluded that a higher 3-hydroxykynurenine/kynurenic acid ratio in plasma could serve as a biomarker in the diagnosis of dyskinesia induced by L-DOPA [42]. In another targeted metabolomic study that compared the levels of some metabolites in PD patients in early Hoehn–Yahr stage ≤ 2 with those in the advanced stage of the disease (Hoehn–Yahr stage > 2), Chang et al. [41] reported that the former category of patients had lower levels of kynurenine acid (KA) and kynurenine acid/kynurenine ratio and higher levels of quinolinic acid (QA) and QA/KA ratio when compared with patients in the early stage of the disease and normal controls, demonstrating significant metabolite signatures that could serve as biomarkers in the plasma of PD patients.

### 3.2. Diabetic Retinopathy

Diabetic retinopathy is one of the major complications that result from chronic, long-standing diabetes mellitus among individuals who suffer from this medical condition. Mazumder et al. [44], in a study involving human subjects, utilised FTIR spectroscopy to analyse serum samples and identified 12 important biomarkers with potentials to significantly discriminate between diabetic patients with retinopathy and those without this complication. Two of these biomarkers were associated with carbohydrate metabolism, 5 with alterations in lipid contents, 4 with protein phosphorylation, and 3 with the amide II group [44]. An untargeted mass spectrometry metabolomic approach was equally applied for the analysis of the vitreous humour of patients with rhegmatogenous retinal detachment, diabetic retinopathy, and healthy subjects in a recent study [45]. Here, significant alterations in glucose metabolism and activation of the hexose monophosphate shunt were reported. In addition, alteration in purine metabolism (characterised by a decrease in xanthine and elevation in purine-related compounds, such as inosine, hypoxanthine, urate, and allantoate) was seen in those with diabetic retinopathy but absent in those with retinal detachment and healthy controls. The researchers concluded that significant perturbations in vitreous humour metabolism in diabetic retinopathy account for the characteristic oxidative stress seen in patients with this complication and show how the vitreous metabolite profile could be affected by the disease [45]. Again, noteworthy diagnostic biomarkers have been identified for proliferative diabetic retinopathy, a leading cause of irreversible blindness in adults with type 2 diabetes mellitus [30]. In a large population-based study aimed at profiling the plasma metabolites of patients with proliferative diabetic retinopathy using liquid chromatography–mass spectrometry, Zhu and others [30] reported alterations in the metabolism of 63 metabolites, out of which 4 metabolites, namely, fumaric acid, uridine, acetic acid, and cytidine (with areas under the curve of 0.96, 0.95, 1.0, and 0.95, respectively), were identified as candidate biomarkers for this sight-threatening condition. According to the researchers, this study was the first to report fumarate as a novel biomarker in relation to diabetes or diabetic retinopathy diagnosis [20]. Using a combination of gas chromatography time-of-flight mass spectrometry and ultraperformance liquid chromatography–quadrupole time-of-flight mass spectrometry tools, Rhee et al. [46] identified plasma glutamine and glutamate as potential biomarkers for predicting the development of diabetic retinopathy in patients with long-standing diabetes. That significant oxidative stress and alteration in the pathway of glutamate metabolism are associated with diabetic retinopathy was equally reported by Jin et al. [47]. Other metabolites that have shown relevance as important biomarkers for making diagnosis or predicting prognosis in diabetic retinopathy include lactic acid, succinic acid, 2-hydroxybutyric acid, asparagine, dimethylamine, histidine, threonine, and glutamine [47].

### 3.3. Alzheimer’s Disease (AD)/Dementia

This is a progressive degenerative disease affecting the brain. It is a leading cause of morbidity and mortality among people with diabetes mellitus and hypertension globally. AD is a major cause of vascular dementia, a very debilitating condition characterised by a progressive decline in memory and behavioural and social skills, especially in the elderly. Presently, biomarkers for early disease diagnosis are inadequate in that they are mostly invasive, time-consuming, and expensive [2]. To this end, a blood-based approach for diagnosing AD using metabolic fingerprinting has been proposed [67]. Metabolomic approaches have shown great promise in bridging this gap, especially with its application on blood samples, a less invasive and cheaper mode of sample collection, in advanced stages (e.g., with dementia) of the disease [68]. In a recent large population study aimed at assessing the association between plasma total tau levels, cognitive decline, and risk of mild cognitive impairment in dementia, Mielke et al. [61] reported that higher total tau levels were associated with significant reduction in cognition, memory, attention, and visuospatial ability of the patients, while this association is independent of a rise in the level of brain amyloid beta (Aβ) peptides. Identification of diagnostic biomarkers in the saliva of individuals with AD has equally been reported using proton-NMR-based metabolomics [20]. In this pilot study, significant concentration changes in the levels of 22 metabolites were observed in the saliva samples of those with mild cognitive impairment and dementia compared with healthy control. The implication of these findings is that, by simply collecting blood samples or saliva from an individual with Alzheimer’s disease, progression to vascular dementia or other cognitive impairment could be detected early easily and managed promptly.

### 3.4. Cardiovascular Disease

Cardiovascular disease (CVD) is a major cause of stroke and sudden death, especially among adults with hypertension. Notwithstanding the several risk managements, preventive measures, and treatment modalities that have been initiated for CVDs, patients have continued to die from cardiac-related complications, hence the need to identify novel therapeutic strategies for managing this condition [69]. Metabolomics has offered a very promising alternative to surmounting this problem by making possible the identification of important biomarkers for diagnosis and risk assessment for CVD development even before patients begin to show overt symptoms [61]. In a recent study involving the use of an untargeted metabolomic approach, namely, stable isotope dilution tandem MS (LC–MS/MS), Li et al. [18] identified trimethyllysine as a predictor of incident cardiovascular risk. Using Spearman’s correlation analyses, the researchers discovered a significant correlation between this biomarker (trimethyllysine) and the artherogenic metabolite trimethylamine *N*-oxide. In another untargeted metabolomic study aimed at finding the association between chronic air pollution exposure and risk of developing bronchial asthma and cardiovascular disease, Jeong and others [48] identified three important pathways of metabolism, namely, linoleate, glycosphingolipid, and carnitine shuttle pathways as key mediators of these health effects among those who were exposed. The strength of this study, which utilised the novel ‘meet in the middle’ statistical approach, lies in the fact that it was prospective in nature (namely, a case–control study nested in longitudinal cohorts) [34]. Metabolomic tools have also been employed to study the pathology of myocardial infarction (MI). Zhu et al. [49] examined the plasma of MI patients using UPLS–Q/TOF–MS and identified 10 metabolites that satisfactorily distinguished MI patients from healthy controls. These metabolite biomarkers include C16-sphingosine, *N*-methyl arachidonic amide, phosphatidylserine, *N*-(2-methoxyethyl) arachidonic amide, linoleamidoglycerophosphate choline, lysophosphatidylcholine (C18:2), lysophosphatidylcholine (C16:0), lysophosphatidylcholine (C18:1), arachidonic, and linoleic acid, as well as perturbations in energy, fatty acid, and phospholipid metabolism. Aside from its potential in discriminating between persons with myocardial infarction and healthy controls, metabolomics has equally been shown to have the ability to predict the risk of developing acute myocardial infarction with fragmented QRS in patients who undergo percutaneous coronary intervention (PCI) [50]. In a recent hospital-based cohort study, Li et al. [50] performed a global metabolic profiling using UPLS–Q/TOF–MS on a cohort of 136 non-coronary artery disease and 118 acute myocardial infarction (AMI) patients undergoing PCI and identified four metabolites as important biomarkers. These metabolites (acetylglycine, threoninyl-glycine, glutarylglycine, and nonanoylcarnitine) were reported to significantly discriminate between AMI with fragmented QRS and that without it. In another study involving 79 patients with heart failure, Chen and others [52] reported that significant perturbations in fatty acid metabolism are associated with acute decompensation in this patient category. In this case–control hospital-based study, acylcarnitine was identified and quantified as an important metabolite biomarker using MS spectroscopy. Serum concentrations of three other metabolites, namely, citrate, tyrosine, and 2- and 3-hydroxybutyrates, have also been linked to increased mortality rate among patients with acute heart failure [55]. Other cardiovascular diseases for which a metabolomic approach has been used include ischaemic cardiomyopathy [51], coronary artery disease [53], ischaemic stroke [54,64], myocardial infarction [56], atherosclerosis [58], and heart failure [64]. Furthermore, metabolomic tools have been used to study the mechanistic pathways involved in the therapeutic effects of some herbal medicines [62].

### 3.5. Inborn Error of Metabolism

Inborn errors of metabolism (IEMs) are a heterogeneous group of hereditary disorders that occur commonly among under-5 children. Traditionally, the diagnosis of IEMs relies on history taking, clinical examination, and a few biochemical tests. The risk of having false negatives with the use of this mode of diagnosis is, therefore, remarkably high, and examining only a limited section of the pathway of metabolism hinders the discovery of novel metabolic disorders [59]. An untargeted metabolomic approach that utilises a single platform rather than targeted metabolite profiling with multiple platforms is now being recommended in that it is cost-effective and helps in identifying a huge amount of small-molecular-weight compounds in a single metabolic pathway [60]. In their recent study, Bonte et al. [60] carried out an untargeted metabolic screening for IEMs using a semiautomatic sample preparation with a UHPLC–Orbitrap–MS platform on 53 patients with 33 distinct IEMs and 260 controls. This novel metabolomic approach identified well over 17,256 compound ions and was able to correctly diagnose IEM in about 90% of cases, including the detection of mannosyl-β1,4-*N*-acetylglucosamine, the latest biomarker for β-mannosidase deficiency [60]. In this study, two diagnoses, however, remained undetected, namely, alkaptonuria and mevalonic aciduria. Except for a few cases of IEMs, such as argininosuccinate lyase deficiency, dimethylglycine dehydrogenase deficiency, and GAMT deficiency, Coene and others [59] were also able to accurately diagnose 42 out of 46 cases using a similar metabolic platform (high-resolution liquid chromatography–quadrupole time-of-flight (LC-QTOF)), underscoring the vital role of metabolomics in identifying disease-specific biomarkers among suspected cases of IEMs.

## 4. Methods

Medline search (linked to medical subject headings—MeSH) conducted on 24 May 2021 for recent studies involving the use of metabolomic tools for biomarker discovery in disease diagnosis and management yielded 453 records (Table 3). Additional 16 records were retrieved from other databases and hand-searched references.

Overall, 26 papers met our inclusion and exclusion criteria. Only original articles that have identifiable metabolites as biomarkers and that report the use of analytical tools such as NMR spectroscopy, MS, and FTIR spectroscopy in any of the five selected disease conditions were included in this study. Other inclusion criteria were the use of a noninvasive (such as the use of urine, sweat, breath) or minimally invasive (such as the use of venepuncture) mode of sample collection. As such, review papers, mini reports, studies in animal subjects, or studies that involved the use of invasive techniques, such as lumbar puncture, instrumentation, or surgery, were excluded. To capture only recent developments in this field, we limited our search to the years 2015–2020. With these inclusion and exclusion criteria in mind, we initially reviewed abstracts of related articles before retrieving the full texts of papers that were considered relevant for our inclusion and assessment. We additionally hand-searched references of studies that were initially accessed for other articles that could be of relevance to our research focus. Finally, only 26 papers were included in this review.

## 5. Research Limitations

One of our research goals was to be able to delineate what profiling approach is utilised in each of the studies included in this review. However, only three [45,48,53] of the identified articles expressly reported utilising untargeted approaches in their studies; others did not report whether their metabolite profiling study was targeted or untargeted (global). Another limitation was paucity of relevant literature, arising from the extremely strict inclusion/exclusion criteria we employed. This makes it extremely hard to draw far-reaching, evidenced-based conclusions on the usefulness of metabolomic tools in disease diagnosis and management.

## 6. Conclusions and Future Perspective

The field of metabolomics, no doubt, has grown and continues to grow beyond merely profiling the metabolites in biological samples to identification of novel biomarkers of disease diagnosis, treatment, progression, and prognosis. While it has provided enormous insights and better characterisation of biological pathways associated with myriads of pathophysiological disturbances occurring in living organisms, a lot of factors still hinder the translation of such research outputs into clinical and industrial applications [70]. The overarching purpose of this review is to both create an awareness on the relevance of metabolomic profiling to medical practice and the need to harness resources (human and material) towards ensuring that laboratory findings (especially as regards biomarker discovery) become translational. In 2018, experts in metabolomics met at an Australian and New Zealand Metabolomics Conference (ANZMET 2018) held in Auckland, New Zealand, where they identified several factors mitigating against the applicability of metabolomic approaches in the clinics [70]. Among others, poor public perception of metabolomics as an important field of ‘omics’ science, costs of procuring analytical instruments, the multidisciplinary nature of metabolomic studies (requiring inputs of experts from different fields of life sciences), and variability in the modes of data acquisition were noted as constituting major bottlenecks in the development of translational metabolomics [70]. Equally significant is the fact that metabolomics does not take into consideration the pivotal role that gender plays as an important determinant of some disease conditions. To ensure better personalised treatment, experts have equally suggested that metabolomic studies must take sex into consideration [10]. The number of metabolites identified as biomarkers for some disease conditions is large, making it difficult to pin down what metabolites are the most important biomarkers or predictors of disease development, progression, or therapeutic response. Future research efforts, therefore, must be geared towards addressing the issue of cost and other challenges that make metabolomics difficult to apply in the clinics. Funding research and developing algorithms that reduce the number of diagnostic, therapeutic, or prognostic biomarkers to the barest minimum should also be vigorously pursued. Finally, we have noted here that the use of a noninvasive or minimally invasive mode of sample collection for biomarker discovery is possible and holds great promise for acceptability by patients, especially when combined with analytical tools with wide metabolite coverage [71]. However, there is a need for the validation and optimization of these tools to arrive at more accurate and precise metabolic biomarkers that are useful in this respect.

## Figures and Tables

**Table 1:** The proportion of analytical tools that were employed in the included studies.

S/No	Analytical Tool	Proportion (%)
1	Mass spectrometry based	65.4
2	NMR spectroscopy based	19.2
3	Others	15.4

**Table 2:** A few metabolic biomarkers of diagnostic and prognostic significance.

S/N	Disease Condition	Metabolic Biomarkers/Pathway	Analytical Platform	Statistics	References
1	Parkinson’s disease	Long-chain acylcarnitine	CE–TOF/MS	ROC	[42]
		Kynurenic acid, quinolinic acid, ratio of kynurenic acid/kynurenine, ratio of quinolinic acid/kynurenic acid	UPLC–TOF/MS	OPLS-DA	[41]
		3-hydroxykynurenine/kynurenic acid ratio	LC–MS	*t*-test	[43]
2	Alzheimer’s disease/ dementia	Total plasma tau			[61]
			^1^H NMR		[20]
3	Diabetic retinopathy	Perturbations in carbohydrate metabolism, lipid contents, biomarkers associated with phosphorylation and amide II group	FTIR spectroscopy	Difference between mean spectra (DBMS), forward feature selection (FFS), and Mann–Whitney *U* tests	[44]
		Alterations in glucose and purine metabolism; activation of the hexose monophosphate shunt	Untargeted MS		[45]
		Fumarate, uridine, acetic acid, and cytidine	LC–MS	Area under the curve (AUC)	[30]
		Plasma glutamine and glutamate	GC–MS/UPLC–MS	Multivariate analysis	[46]
		Activation of alanine, aspartate, and glutamate metabolic pathways	NMR-based spectroscopy	PCA, heat map analysis, average change analysis	[47]
4	Cardiovascular disease	*N*6,*N*6,*N*6-trimethyl-L-lysine	Stable isotope dilution tandem MS (LC–MS/MS)	Spearman’s correlation analyses	[18]
		Linoleate metabolism, glycosphingolipid metabolism, and carnitine shuttle pathway	Untargeted metabolomics	‘Meet in the middle’ statistics	[48]
		Acetylglycine, threoninyl-glycine, glutarylglycine, and nonanoylcarnitine	UPLS–Q/TOF–MS	ROC with AUC, sensitivity, specificity	[62]
		Phosphatidylserine, C16-sphingosine, *N*-methyl arachidonic amide, *N*-(2-methoxyethyl) arachidonic amide, linoleamidoglycerophosphate choline, lyso-PC (C18:2), lyso-PC (C16:0), lyso-PC (C18:1), arachidonic acid, and linoleic acid	UPLS–Q/TOF–MS	PCA, PLS-DA	[49]
		*N*8-acetylspermidine	LC–FT spectroscopy MS	Student *t*-test, ANOVA, Mann–Whitney *U* test, Kruskal–Wallis test, chi-square	[51]
		Acylcarnitine	MS	Paired *t*-test, generalised estimating equations	[52]
		Urea cycle/amino group, tryptophan, aspartate/asparagine, lysine, tyrosine, and carnitine shuttle pathways	LC–MS	*t*-test, chi-square	[53]
		Asparagine, tyrosine, xylose, for ischaemic stroke	LC–MS	Wilcoxon test, OPLS-DA	[54]
		Sphingomyelin for incident ischaemic stroke	LC–MS	Paired Wilcoxon rank test	[63]
		Citrate, tyrosine, 2- and 3-hydroxybutyrates for acute heart failure	NMR spectroscopy	Logistic regression analysis	[55]
		23 metabolites, with higher levels of 7 (3-hydroxybutyrate, proline, acetate, creatinine, acetone, formate, mannose) and lower levels of 2 (valine, histidine) as predictors of mortality	NMR spectroscopy	ROC, multivariate regression/PCA, Cox models	[56]
		104 metabolites, with lower levels of 7 (pelargonic acid, glucosamine/galactosamine, thymine, 3-hydroxybutyric acid, creatine, 2-aminoisobutyric acid, hypoxanthine) as correlates for coronary artery disease	CE–TOF/MS	Unsupervised PCA	[57]
		2-Hydroxycaproate, gluconate, and sorbitol for atherosclerosis	UPLC–MS	ROC	[58]
		13 metabolites, 2 of which (phenylalanine and acetate) were significant predictors of heart failure hospitalisation	NMR spectroscopy	*t*-test, Cox proportional hazard regression	[64]
5	Inborn errors of metabolism	Mannosyl-β1,4-N-acetylglucosamine, the biomarker for β-mannosidase deficiency; correctly diagnosed 90% of IEM cases	UHPLC–Orbitrap–MS	*Z*-scores	[60]
		Correctly identified 42 out of 46 IEM cases	LC–QTOF–MS	Two-sided *t*-tests	[59]

**Table 3:** Summary of the MeSH search terms conducted for the retrieval of recent studies in metabolomics.

#	Searches	Results
1	exp metabolomics/ or exp lipidomics/	19,211
2	exp biomarkers, pharmacological/	1963
3	Diagnosis/	17,434
4	Prognosis/	533,501
5	Therapeutics/	8516
6	2 or 3 or 4 or 5	559,030
7	1 and 6	453
